# *Fasciola hepatica* Surface Coat Glycoproteins Contain Mannosylated and Phosphorylated N-glycans and Exhibit Immune Modulatory Properties Independent of the Mannose Receptor

**DOI:** 10.1371/journal.pntd.0004601

**Published:** 2016-04-22

**Authors:** Alessandra Ravidà, Allison M. Aldridge, Nicole N. Driessen, Ferry A. H. Heus, Cornelis H. Hokke, Sandra M. O’Neill

**Affiliations:** 1 Parasite Immune Modulation Group, School of Biotechnology, Faculty of Science and Health, Dublin City University, Glasnevin, Dublin, Ireland; 2 Department of Parasitology, Leiden University Medical Center, Leiden, The Netherlands; McGill University, CANADA

## Abstract

Fascioliasis, caused by the liver fluke *Fasciola hepatica*, is a neglected tropical disease infecting over 1 million individuals annually with 17 million people at risk of infection. Like other helminths, *F*. *hepatica* employs mechanisms of immune suppression in order to evade its host immune system. In this study the N-glycosylation of *F*. *hepatica’s* tegumental coat (FhTeg) and its carbohydrate-dependent interactions with bone marrow derived dendritic cells (BMDCs) were investigated. Mass spectrometric analysis demonstrated that FhTeg N-glycans comprised mainly of oligomannose and to a lesser extent truncated and complex type glycans, including a phosphorylated subset. The interaction of FhTeg with the mannose receptor (MR) was investigated. Binding of FhTeg to MR-transfected CHO cells and BMDCs was blocked when pre-incubated with mannan. We further elucidated the role played by MR in the immunomodulatory mechanism of FhTeg and demonstrated that while FhTeg’s binding was significantly reduced in BMDCs generated from MR knockout mice, the absence of MR did not alter FhTeg’s ability to induce SOCS3 or suppress cytokine secretion from LPS activated BMDCs. A panel of negatively charged monosaccharides (i.e. GlcNAc-4P, Man-6P and GalNAc-4S) were used in an attempt to inhibit the immunoregulatory properties of phosphorylated oligosaccharides. Notably, GalNAc-4S, a known inhibitor of the Cys-domain of MR, efficiently suppressed FhTeg binding to BMDCs and inhibited the expression of suppressor of cytokine signalling (SOCS) 3, a negative regulator the TLR and STAT3 pathway. We conclude that *F*. *hepatica* contains high levels of mannose residues and phosphorylated glycoproteins that are crucial in modulating its host’s immune system, however the role played by MR appears to be limited to the initial binding event suggesting that other C-type lectin receptors are involved in the immunomodulatory mechanism of FhTeg.

## Author Summary

Fascioliasis, caused by the liver fluke *Fasciola hepatica*, is a neglected tropical disease infecting over 1 million individuals annually with 17 million people at risk of infection. These worms infect the liver and can survive for many years in its animal or human host because they supress the host’s immune system that is important in clearing worm infection. Worms are similar to humans in that they are made of proteins, fats and sugars, and while there are many studies on worm proteins, few studies have examined the sugars. We are interested in the sugars because we believe that they help the parasite survive for many years within its host. To examine this, we have used a technique called mass spectrometric analysis to characterise the sugars present in *F*. *hepatica*. We also have developed systems in the laboratory to test if these sugars can suppress the host’s immune system. We conclude that *F*. *hepatica* sugars are crucial in suppressing its host’s immune system; however, the exact way the sugars can do this requires further studies. These studies are important for the development of worm vaccines or therapies.

## Introduction

Infection with parasitic worms (helminths) modulates the host immune system by biasing T helper (Th) cells towards a Th-2/Treg immune response [1,2] while simultaneously impairing pro-inflammatory Th1/Th17 immunity. This polarisation is due to the interaction of helminth derived molecules with pattern recognition receptors on innate immune cells such as dendritic cells, macrophages and mast cells that drive the polarisation of T-cells [3]. Many helminth proteins and lipids are glycosylated, and the initial interaction of these molecules with innate immune cells is mainly *via* C-type lectin receptors (CLRs) [4,5)]. Helminth-derived glycoconjugates, i.e. *N-* and *O-*glycoproteins and glycolipids, often contain a mixture of glycan motifs similar or identical to those present in the host, and structurally distinct pathogen-related motifs [6].

With respect to structural analysis of parasitic helminth glycans, *Schistosoma mansoni* has been most extensively studied [7,8]. Although glycan profiles are extremely complex and specific for each life-stage or preparation (e.g. excreted *vs*. somatic antigens), common features of schistosome glycans are the presence of a high proportion of Lewis X elements, *N*,*N’-*diacetyllactosamine motifs (LacdiNAc; LDN) α1–3 substituted with single Fuc or unique Fucα1-2Fuc difucosyl units, and N-glycan core modifications with xylose and fucose [7,8,9,10,]. While in most cases both glycans and proteins have a role to play [11,12,13,14,], some isolated glycan structures have been proven to directly activate host cells when presented as multivalent arrays on carrier proteins [15].

Several reports have pointed to a role for CLRs in mediating immune regulatory processes driven by helminth-derived glycoconjugates [11,16,17]. *Schistosoma mansoni* soluble egg antigens signal through several CLRs, dendritic cell-specific intercellular adhesion molecule-3-grabbing non-integrin (DC-SIGN), Macrophage galactose lectin (MGL) and Mannose receptor (MR), inhibiting dendritic cells maturation and influencing the development of Th2 immune response. Similarly, *Trichuris suis* antigens signal through MR and DC-SIGN which are involved in inhibiting a pro-inflammatory dendritic cell phenotype [17]. Recent studies demonstrated the direct interaction of MR with *Fasciola hepatica* and specific *S*. *mansoni* derived antigens [11,18].

*Fasciola hepatica* is a parasitic flatworm that infects humans and livestock worldwide. The economic burden of *F*. *hepatica* infection to the agricultural industry is estimated at $3 billion per year [19] while an estimated 1 million people are infected worldwide [20,21]. *F*. *hepatica* shares with other helminths [22,23,24] the ability to polarise Th2 immune responses within hours post infection while simultaneously impairing the ability of innate immune cells to promote Th1/Th17 immune responses [25,26]. We are interested in the tegumental antigens (FhTeg) that are released continuously by *F*. *hepatica* during infection and exposed to host immune responses. FhTeg exhibits potent Th1 immune suppressive properties *in vivo* by suppressing serum levels of the Th1 mediators IFNγ and IL-12p70 in the mouse model of septic shock [27]. FhTeg-activated dendritic cells and mast cells are hypo-responsive to TLR activation thereby suppressing the production of inflammatory cytokines and co-stimulatory molecules important in driving adaptive immune responses [27,28]. FhTeg mechanism of action is independent of TLRs and has been linked to the suppression of NF-κB and MAPK pathway [29] and enhanced expression levels of suppressor of cytokine signalling (SOCS) 3, a negative regulator of the TLR and STAT3 pathway [30]. More recently it was shown that SOCS3 expression was enhanced in the liver of infected mice [31].

FhTeg is a biological matrix rich in glycoconjugates that remains continually exposed to the host immune system and is unique to each *Fasciola* species [32]. While proteomic analyses confirm the high abundance of glycoproteins in the tegument preparation [33,34], detailed glyco-analytical data are available only for the glycolipid fractions of *Fasciola* spp. [35,36,37]. With a view to further understanding *F*. *hepatica*’s mechanism of immune suppression, in this study we investigated the *N-*glycosylation profile of FhTeg and explored the role played by oligomannose and negatively charged glycans in immunomodulatory mechanism of FhTeg.

## Materials and Methods

### Antigen preparation

FhTeg was prepared as previously reported [27]. In brief, *F*. *hepatica* adult worms following collection from sheep at a local abattoir were washed in sterile phosphate-buffered saline (PBS) and incubated in 1% Nonidet P-40 (NP-40 [Sigma Aldrich] in PBS) for 30 min. Supernatant was collected and NP-40 removed using BIO-RAD detergent-removing biobeads (BIO-RAD), and the remaining supernatant was centrifuged at 14,000 × *g* for 30 min at 4°C prior to being filtered/concentrated using compressed air, and then stored at −20°C. All protein concentrations were determined using a bicinchoninic acid (BCA) protein assay kit (Pierce, Fischer Scientific, Dublin, Ireland) and all antigen tested for endotoxin using a using the Pyrogene endotoxin detection system (Cambrex). FhTeg gave endotoxin levels similar to background levels and were less than the lower limit of detection in this assay (<0.01 EU/ml).

### FhTeg enzymatic digestion, *N*-glycan isolation, derivatization and mass spectrometric analysis

*N-*glycans were isolated by sequentially digesting FhTeg-derived glycopeptides with PNGase F and PNGase A (Sigma-Aldrich, Ireland), as previously described [38]. The purified *N*-glycans were subjected to labelling with 2-aminobenzoic acid (2-AA) using a previously described protocol [39]. 2-AA-labeled *N*-glycans were analyzed by matrix-assisted laser-desorption ionization (MALDI) time-of-flight (TOF) mass spectrometry (MS) with an Ultraflex II MALDI-TOF-MS instrument (Bruker Daltonics; Bremen, Germany) operating in the negative-ion reflector mode using DHB (Bruker Daltonics) as a matrix. Aliquots of FhTeg 2-AA-labeled-*N*-glycans were incubated with Jack bean α-mannosidase, neuraminidase from *Vibrio cholera*, β-*N-*acetyl glucosaminidase from *Canavalia ensiformis* (all from Sigma-Aldrich, Wicklow, Ireland), or Jack bean β-(1–4,6) galactosidase (Prozyme-Glyko, Hayward, CA, USA) and analyzed by MALDI-TOF-MS. MALDI-TOF-MS spectra were annotated in terms of monosaccharide composition (F_*x*_H_*y*_N_*z*_) applying the Glyco-Peakfinder tool [40], followed by manual interpretation in-line with the exoglycosidase treatment results, and LIFT fragmentation analysis of selected ion species, using Bruker Daltonics FlexAnalysis software (Bruker Daltonics). All glycan signals were detected as [M-H]^-^. Fourier transform ion cyclotron resonance mass spectrometry (FT-ICR-MS) was performed on a Bruker 12T solariX XR high-resolution MALDI-FT-ICR-MS instrument equipped with a ParaCell (Bruker Daltonics, Bremen, Germany). The system was controlled by ftmsControl software and equipped with a Smartbeam-II laser system operating at 200 Hz. The glycan sample was spotted onto a MALDI ground steel target plate (Bruker Daltonics) in 1 μL water and mixed on plate with 1μL super-DHB (Sigma-Aldrich) (2.5 mg/mL solution in acetonitrile/water, 1:1 containing 1mM NaOH).

### Lectin and western blots

Biotinylated Concanavalin A lectin (ConA) used in this study was purchased from Vector Laboratories (Peterborough, UK). It was selected as the plant lectin with highest affinity for mannose-rich carbohydrate epitopes. IRDye 800 Streptavidin was purchased from Li-COR Biosciences (Lincoln, MA, USA). Precast 4–20% sodium dodecyl sulphate (SDS)-polyacrylamide gels (Pierce) were run under standard conditions (see supplementing material). The gels were transferred onto nitrocellulose membranes by iBlot Dry blotting system (Invitrogen, Carlsbad, CA, USA). After standard western blotting procedure, the membranes were scanned using Odyssey Infrared Imaging System (Li-COR Biosciences). Data analysis was performed with Odyssey V 3.0 software (Li-COR Biosciences).

### Lectin-fluorescence microscopy of whole flukes

The following protocol was adapted from a previously described method [41]. Briefly, adult liver flukes were flat-fixed in 4% paraformaldehyde and incubated with fluorescein-labelled ConA (Vector Laboratories). Parasites were mounted on glass microscope slides with Vectashield anti-fading solution (Vector Laboratories). Specimens were viewed using a Leica DM IL LED microscope using 10x, 20x, and 40x HI PLAN I objectives (Leica Microsystems, Wetzlar, Germany) equipped with epifluoresce source and a filter system for FITC fluorescence. Images were processed with Adobe Photoshop CS4 software (Adobe System Inc., San Jose, USA).

### Ethics statement

BALB/C mice, 6–8weeks old (Charles River, Carrentrilla, Ireland), were kept under specific pathogen-free conditions at the Bioresource Unit, Faculty of Health and Science, Dublin City University (DCU), Ireland. All mice were maintained according by European Directive 2010/63/EU. Ethical permission for the use of animals and experimental protocols were approved by the Health Products Regulatory Authority, Ireland (licence number B100/2833) and Dublin City University Ethics committee (reference number: DCUREC/2010/033). We adhered to all agreed protocols.

### Cell culturing

Bone marrow-derived immature DCs were isolated from the femurs and tibia of BALB/C mice according to Lutz *et al*. protocol [42], yielding >95% pure BMDC population (on the basis of CD11c expression). The same protocol was also performed on C57/BL mice or from MR knockout mice on a C57/BL background (a gift from Professor Padraic Fallon, Trinity College Dublin). CHO cell line stably expressing murine MR [43] and untransfected controls were maintained in RPMI 1640 Glutamax (Gibco, Life Technologies, Bleiswijk, Netherlands) containing 10% foetal calf serum (FCS; Bodinco B. V., Alkmaar, Netherlands). All media were supplemented with penicillin (Astellas Pharma B.V.) and streptomycin (Sigma-Aldrich), and transfected cell lines were continuously kept under selection of 0.6 mg ml^−1^ geneticin (Gibco).

### Cellular adhesion assays

Cellular adhesion assay was performed as previously reported [43]. Briefly, FhTeg and BSA were fluorescently labelled with PF-647 or PF-488 using the Promofluor labelling kits according to the manufacturer’s recommendations (Promokine, Heidelberg, Germany). Where indicated cells were pre-incubated with EGTA (10mM, Sigma-Aldrich), anti-MR (1 μg ml^-1^, clone: 15–2, Abcam, Cambridge, UK), mannan (0.1–1 mg ml^−1^, Sigma-Aldrich), GalNAc-4S (1-25mM, Sigma-Aldrich), for 45 min at 37°C prior to addition of fluorescently labelled FhTeg in the stated concentrations at four degrees. As control for non-specific binding, cells were incubated with fluorescently labelled BSA at four degrees. After extensive washes, binding was analysed by flow cytometry (BD FACSAria or FACSCanto, BD Biosciences), using FacsDiva (BD Biosciences) and FlowJo Software (TreeStar, Ashland, OR, USA).

For microscopy studies, after the final incubation, cells were washed with PBS and fixed in 4% paraformaldehyde. After extensive rinsing, cells were resuspended in Vectashield anti-fading solution with DAPI (Vector Laboratories), mounted on slides and viewed using a Leica DM IL LED microscope as described above.

### RNA extraction and qPCR

BMDCs were stimulated with or without GalNAc-4S (1mM), Mannan (50 μg ml^-1^) or Man-6P (50 μg/ml), 30 min prior to stimulation with FhTeg (10 μg ml^-1^). Cells were harvested after 2.5 h and washed before RNA extraction with high pure RNA isolation kit (Roche Diagnostics, Burgess Hill, UK) according to the manufacturer’s instructions. cDNA was synthesized using a reverse transcriptase kit (Roche) according to the manufacturer’s protocol. The quantitative PCR (qPCR) transcription analysis was carried out on a real-time thermal cycler LightCycler96 (Roche), using Real Time Ready primers (Roche) specific for SOCS3 and three internal reference genes: β-actin (NM_007393.3), GAPDH (NM_008084.2), and Gusb (β-glucuronidase, NM_010368.1). The Pfaffl method was used to calculate fold changes compared to the three reference genes.

### BMDCs stimulation and cytokine secretion analysis

BMDCs were stimulated with FhTeg (10 μg/ml) for 2.5 h prior to stimulation with LPS (Escherichia coli R515; 100 ng ml^-1^; Enzo Life sciences, Farmingdale, USA), for 18 h. Alternatively, cells were preincubated with GalNAc-4S (10 mM), Mannan (500 μg ml^-1^) or Man-6P (50 μg/ml) for 30 min prior to stimulation with *F*. *hepatica* antigens. Control cells were treated with medium, FhTeg antigens (also in combination with carbohydrate inhibitors) or LPS alone. Supernatants from cultured DCs were tested for the production of IL-12p70, and TNF (BD OptEIA ELISA sets; BD Biosciences).

### Statistics

All data were analysed for normality prior to statistical testing by Prism 6.0 (GraphPad Software Inc, La Jolla, CA, USA) software. Where multiple group comparisons were made, data were analysed using one-way ANOVA. For comparisons between two groups, the Student’s *t* test was used. In all tests, *p* < 0.05 was deemed significant.

## Results

### FhTeg preparation is rich in oligomannose glycans and contains truncated complex type *N-*glycans with subsets substituted with negatively charged groups or α1-6-linked core fucosylation

**Fig 1:**
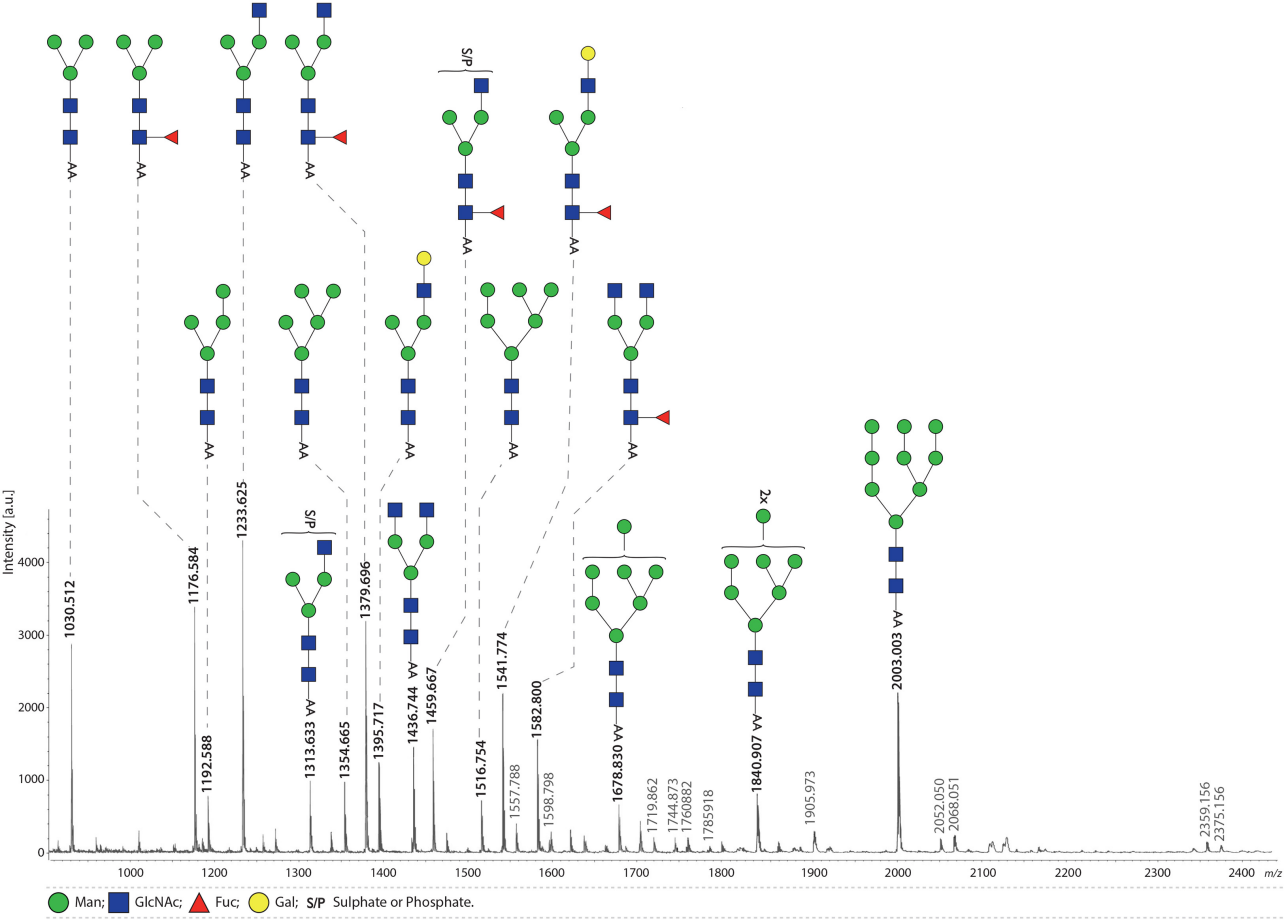
FhTeg preparation is rich in oligomannose and truncated complex type *N-*glycans carrying fucose or sulfate/phosphate moieties. Fh tegumental antigens were digested with trypsin followed by PNGase F treatment. Released N-glycans were subsequently labelled with 2-AA and analysed by MALDI-TOF-MS in the negative ion-reflector mode. Signals are labelled with monoisotopic masses. Most abundant *N*-glycan structures are annotated in the spectrum while minor peaks are reported in the supplementing material (S1 Table). The signal at *m/z* 1582.8 [M-H]^-^ is annotated according to MALDI-TOF/TOF-MS analysis.

**Fig 2:**
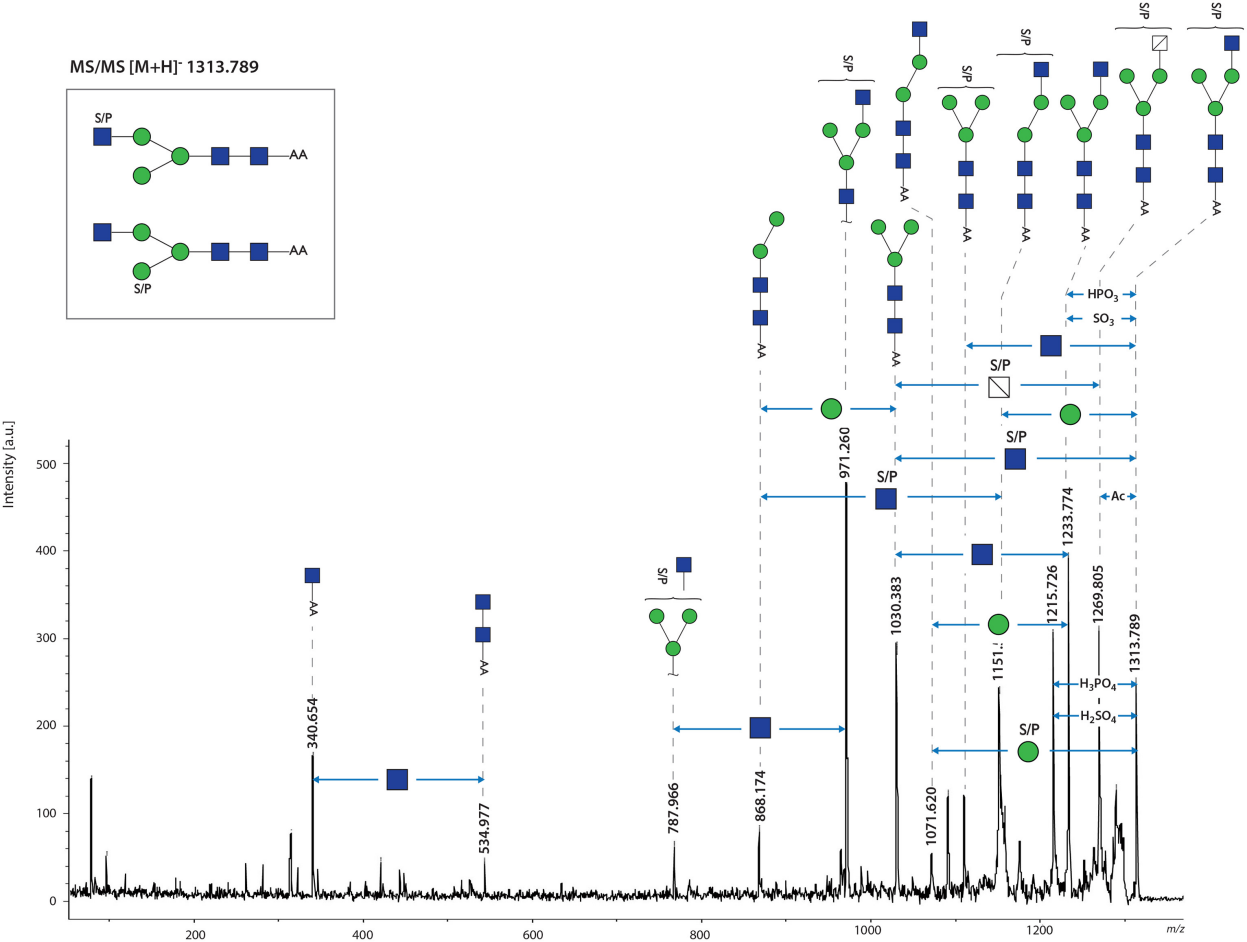
FhTeg *N-*glycans carry core fucose and are sulphated/phosphorylated on terminal *N*-acetylglucosamine and mannose residues. The sulphated/phosphorylated glycan observed at *m/z* 1313.6 [M-H]^-^ in Fig 1 was analysed by MALDI-TOF- MS/MS. Differences in monosaccharides and functional groups content are indicated by double headed arrows.

This study is the first to present the MS analysis of FhTeg derived N-glycans. The spectrum of PNGase-F released glycans contains major signals indicative of oligomannose type structures (Man_4-9_GlcNAc_2_, *m/z* 1192.6, 1354.7, 1516.8, 1678.8, 1840.9, 2003.0 [M-H]^-^, respectively) (Fig 1), trimannosyl glycans with and without α1–6 core fucose (Fuc) (*m/z* 1176.6, 1030.5, respectively), and complex type glycans with single or truncated antenna (*m/z* 1233.6, 1395.7, 1436.7) and their core α1-6-fucosylated variants (*m/z* 1379.7, 1541.8, 1582.8). In addition, relatively abundant signals were observed at *m/z* 1313.6 and 1459.7, which indicate the occurrence of a sulphate/phosphate modified form of the truncated monoantennary glycans. These assignments, based on monosaccharide composition, were fully supported by exoglycosidase digestions (S1A–S1C Fig), and LIFT MS/MS fragmentation analysis of selected ions *m/z* 1313.8, 1459.9, *m/z* 1541.9, 1582.9 (Fig 2). In each glycan the number of antenna was confirmed by removing accessible mannoses with α-mannosidase treatment (S1A Fig). Similarly, the presence of the terminal, non-substituted Gal and/or GlcNAc residues was confirmed by β-hexosaminidase and β-galactosidase treatments (S1B and S1C Fig), and these incubations also confirmed location of Fuc (if present) exclusively on the core GlcNAc. Notably, the sulphated/phosphorylated ions *m/z* 1313.8 and 1459.9 were retained after β-hexosaminidase digestion (S1B Fig). This suggests that terminal GlcNAc residues are substituted with the anionic group, with core fucosylation on peak *m/z* 1459.9.The MS/MS fragmentation however indicates that the S/P group can be present on both the terminal GlcNAc as well as the terminal Man residue (Fig 2). The latter observation is confirmed by the α-mannosidase treatment, which does not remove the substituted mannose (*m/z* 1459.6 in S1A Fig). The spectrum of PNGase A released glycans didn’t reveal any additional peaks if compared to PNGase F digestion (S2 Fig), thus suggesting the absence of core α1-3-fucosylated glycans.

Minor peaks were also annotated and depicted in supplementing material, S1 Table. Notably, minor signals matching with *N-*acetylneuraminic acid (NeuAc) or *N-*glycolylneuraminic acid (NeuGc) containing N-glycans were detected (S1 Table). In particular, two signals at *m/z* 2052.05 and 2068.05 correspond to sialylated diantennary glycans bearing one NeuAc and one NeuGc, respectively; while signals at *m/z* 2359.2 and 2375.2 correspond to disialylalted glycans. The presence of these capping sugars was validated by neuraminidase digestion for the signals at *m/z* 2052.050 and 2068.0 (S1D Fig).

The presence of sialylated glycoproteins in *F*. *hepatica* has been reported in scattered studies using different experimental techniques [37]. However, since other helminths lack the biosynthetic machinery required for sialylation, the sialic acid-containing glycans in the liver fluke might be derived from host glycoproteins (such as sialylated antibodies), as observed for other parasites [38].

### Mannose-rich glycans of *F*. *hepatica* tegumental antigen are predominantly expressed on the spines and suckers of adult flukes

**Fig 3:**
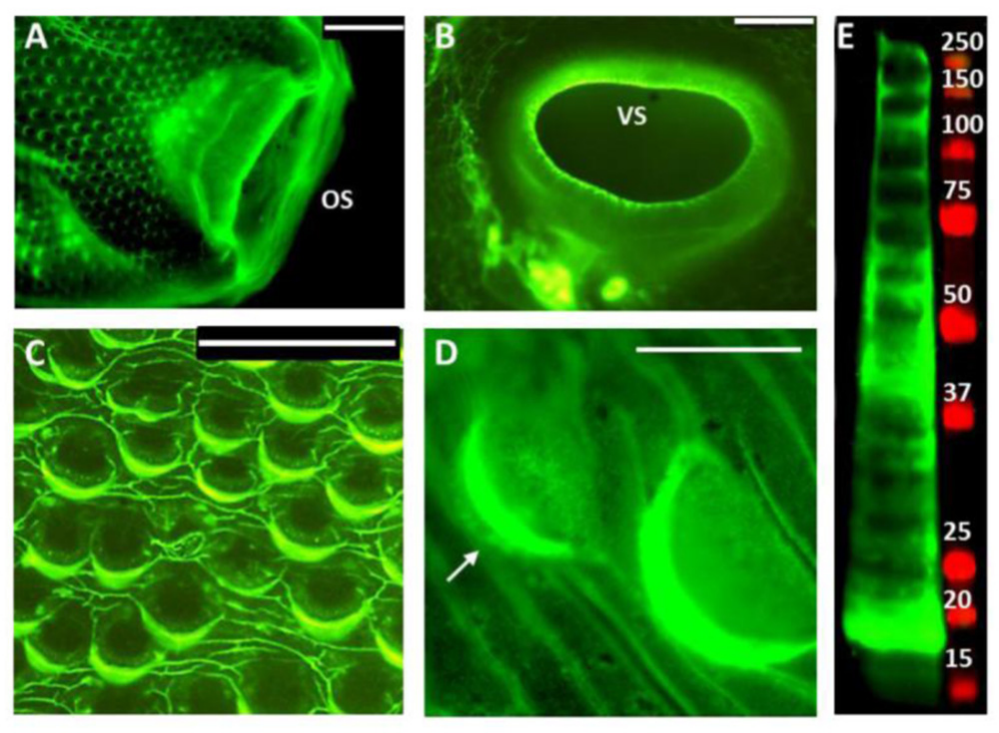
Mannose-rich oligosaccharides are predominantly localized on scales and suckers of adult flukes. High mannose type *N-*glycans are highly expressed on FhTeg as visualised in ConA fluorescence micrographs (A-D) and ConA blot (E) of adult *Fasciola hepatica* tegumental coat. **A-D:** Oral (OS) and Ventral (VS) suckers (A and B, respectively), tegumental spines (C-D) probed with fluorescein-labelled ConA (green); Scale bars: 200μm (A-B), 100μm (C), 50μm (D); spinelets details highlighted with arrows. **E:** Tegumental proteins (15μg) probed with biotin-labelled ConA/IR-labelled streptavidin complex (green), MW marker (Red).

Since high mannose glycans were abundantly present in the N-glycan profile of FhTeg, we incubated flat fixed adult flukes with fluorescein-conjugated ConA, a plant lectin with high affinity for oligomannose type glycans that bind to the carbohydrate-recognition domains on MR (CRD) in order to confirm the presence of oligomannose N-glycans on the surface of *F*. *hepatica* coat. ConA bound to the external morphological features of the adult fluke such as the oral and ventral suckers, tegumental spines and the underlaying tegumental coat (Fig 3A–3D). The abundant expression of glycan motifs on the tegumental spines enabled the visualisation of spinelets, features usually observed only by scanning electron microscopy [44]. In the lectin blot (Fig 3E), ConA recognised a wide array of FhTeg glycoproteins (>15 bands) with electrophoretic mobility ranging between 260 and 17 KDa, with the most intense protein bands at approximately 18 and 40 KDa.

### FhTeg is bound by MR-transfected CHO cells and the interaction can be blocked by sugars that can bind to CRDs and CR-MR domains on the mannose receptor

**Fig 4:**
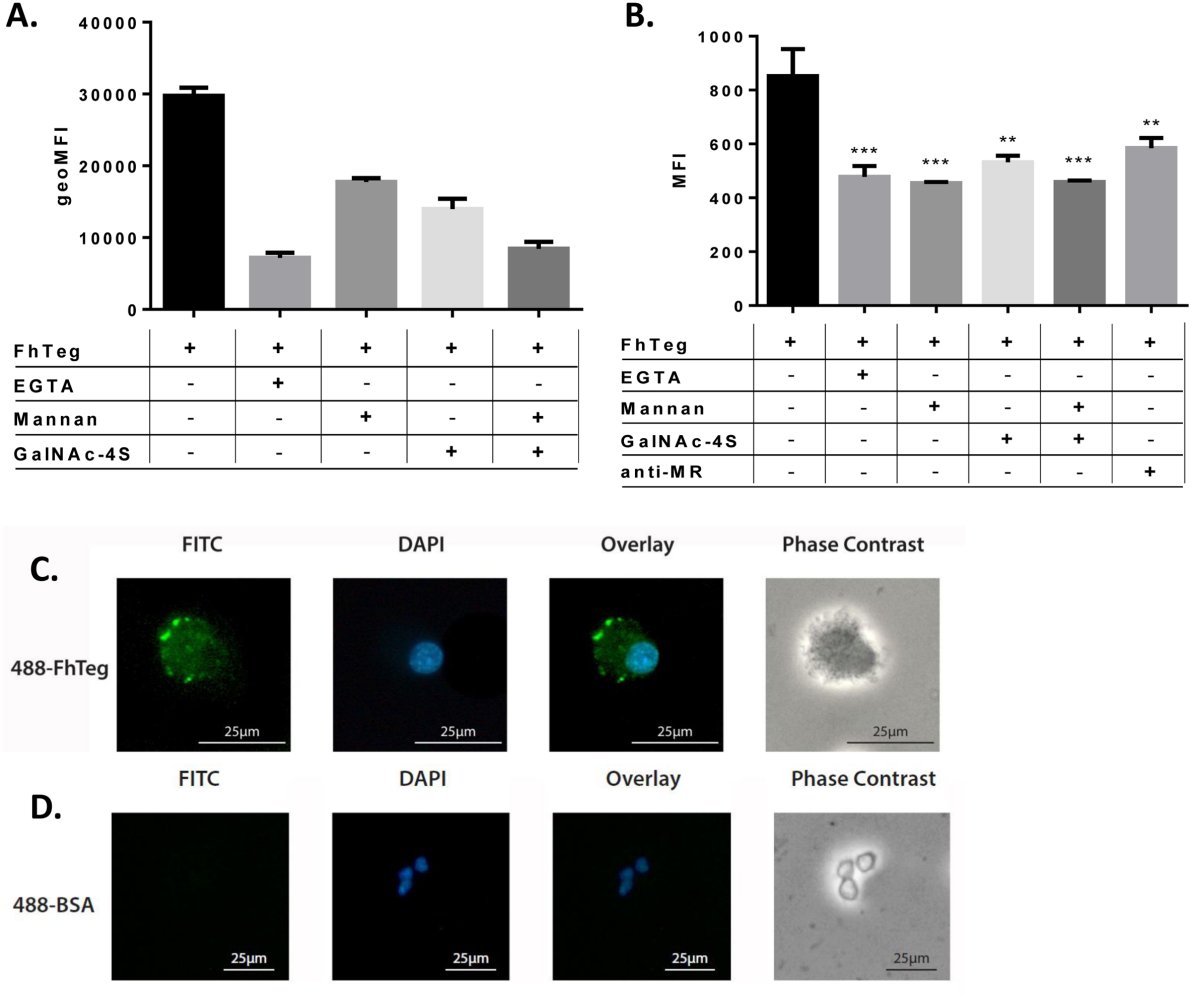
FhTeg binding to dendritic cells is mediated by MR and is carbohydrate and calcium dependent. **A-B:** MR-transfected CHO cells (A) and BMDCs (B) were stimulated with and without inhibitors, i.e. EGTA (10mM), anti-MR (1 μg ml^-1^), mannan (A: 100 μg ml^-1^;B: 1 mg/mL), GalNAc-4S (A: 1mM; B: 25 mM), for 45 min prior to stimulation with fluorescently labelled FhTeg (A: 1–10 μg ml^-1^; B: 5 μg/mL) for 45 min. Fluorescently labelled BSA was also used as control. FhTeg binding to cells was assessed by flow cytometry and reported in bar chart format. Data shown is the mean ± SD of one representative experiment; the experiment was repeated 2–3 times, **, *p* ≤ 0.01; ***, *p* ≤ 0.001 compared to FhTeg. **C-D:** BMDCs were stimulated with fluorescently labelled FhTeg (10μg ml^-1^, green) or BSA (10g ml^-1^, green)) for 45 min prior to paraformaldehyde fixation and mounting with DAPI (blue); Scale bar: 25μm.

To study the potential interaction between FhTeg and MR, the binding of FhTeg by MR-transfected CHO cells was investigated in presence and absence of EGTA and two carbohydrate MR-binding inhibitors, mannan and GalNAc-4S that interact with the CRD (carbohydrate-recognition domains) and CR-MRdomain (*N-*terminal cysteine-rich domain) on MR, respectively [45]. As measured by flow cytometry, FhTeg significantly bound to CHO cells expressing MR compared to untransfected s CHO cells (S2A Fig). This binding was reversed in the presence of EGTA and a combination of mannan and GalNAc-4S (Fig 4A), suggesting the potential involvement of both carbohydrate binding domains in FhTeg-MR interaction.

Given the apparent abundance of mannose (for CRDs) and the potential presence of sulphated GlcNAc (for CR-MR), on the surface of adult flukes, we also examined the interaction of the FhTeg with BMDCs. Both flow cytometry and microscopy analyses (Fig 4B and 4C) revealed that FhTeg strongly adheres to BMDCs compared to fluorescently labelled BSA which was used as a negative control (Fig 4D). Flow cytometric analysis revealed that this interaction is mediated by calcium as the binding was significantly reduced upon pre-incubation with EGTA (p≤ 0.001). The involvement of MR was assessed by pre-incubation of cells with anti-MR blocking antibody or the carbohydrate inhibitors (mannan and GalNAc-4S) prior to stimulation with FhTeg. Pre-incubation of BMDCs with all inhibitors significantly reversed the binding (Fig 4B), implying the involvement of CLRs. This indicates that MR could contribute to FhTeg binding to BMDCs, however it is acknowledged that a variety of other CLRs (e.g. Dectin-1, DC-SIGN, MGL etc) may also be involved.

### The MR receptor does not have a role to play in the induction of SOCS3 or the in suppression of cytokines from TLR activated DCs

**Fig 5:**
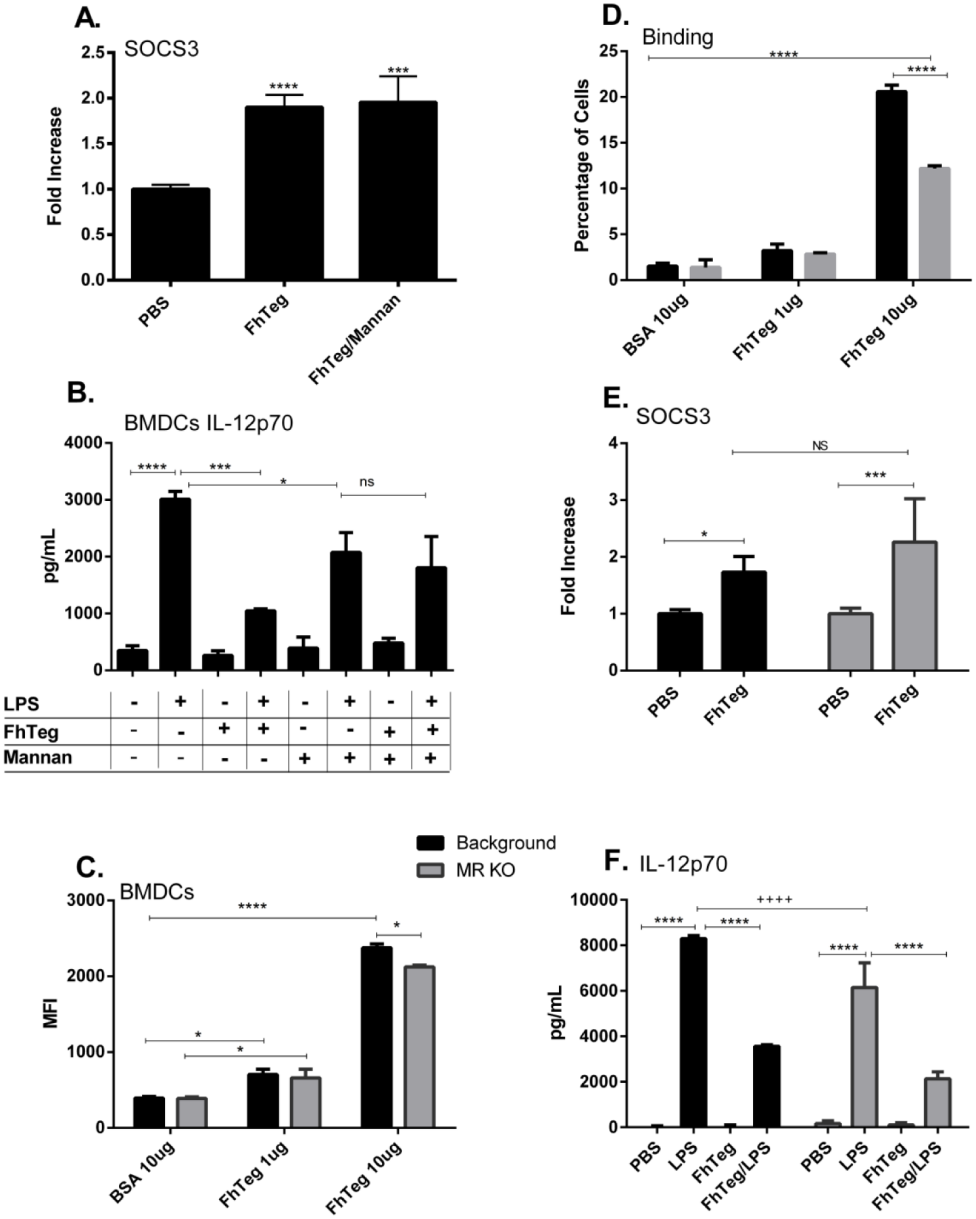
The immune properties of FhTeg are independent of the MR receptor. **A**: BMDCs were stimulated with mannan for 30 min prior to incubation with FhTeg for 2.5 h. Total RNA was extracted, and after reverse transcription cDNA was analyzed with qPCR for SOCS3. RNA expression was normalized to GAPDH and actin control genes. **B:** BMDCs were pre-incubated with mannan prior stimulation with PBS or FhTeg (10μg) before addition of LPS (100ng ml^-1^) for 18 h. IL12p70 levels were measured with commercial ELISA kits. Data are presented as the mean ± SEM of two independent experiments. ***p ≤ 0.001; ****p ≤ 0.0001 compared to LPS group. **C,D:** BMDCs isolated from MR-knockout mice were stimulated with fluorescently labelled FhTeg or BSA (10μg ml^-1^, green) for 45 min prior to paraformaldehyde fixation. FhTeg binding to cells was assessed by flow cytometry and reported in bar chart format. **E:** BMDCs isolated from MR-knockout mice were stimulated with FhTeg (10μg) for 2.5 h. Total RNA was extracted, and after reverse transcription cDNA was analyzed with qPCR for SOCS3. RNA expression was normalized to GAPDH and actin control genes. **F.** BMDCs derived from MR knockout mice were stimulated with **PBS** or FhTeg (10μg) before addition of LPS (100ng ml^-1^) for 18 h. IL12p70 levels were measured with commercial ELISA kits. Data are presented as the mean ± SEM of two independent experiments. *p ≤ 0.05, **, *p* ≤ 0.01; ***, *p* ≤ 0.001.

In previous studies we demonstrated that FhTeg up-regulates SOCS3 in DCs and mast cells *in vitro* [29]. SOCS3 [29] is an intracellular protein regulating the duration or intensity of cytokine-induced signal *via* a negative feedback inhibition mechanism [46]. SOCS proteins are induced *via* JAK/STAT signaling and, among other signals, stimulation of TLRs [47]. Here we determine if the induction of SOCS3 RNA can be inhibited by the addition of mannan to DCs in culture prior to stimulation with FhTeg (10 μg ml^-1^). Notably, incubation with mannan did not alter SOCS3 expression (Fig 5A). Another property of FhTeg is its ability to suppress the LPS-induced production of pro-inflammatory cytokines in BMDCs [27]. Pre-incubation of BMDCs with mannan prior to LPS and FhTeg stimulation reversed IL12p70 secretion (Fig 5B).

To clarify the role of MR in FhTeg-BMDCs interactions, we obtained BMDCs from MR knockout mice. The absence of MR resulted in a statistically significant decrease in the binding of FhTeg to the surface of BMDCs (Fig 5C and 5D). However in the absence of MR, FhTeg retained the ability to induce the expression of SOCS3 (Fig 5E), and to suppress LPS induced IL12p70 expression in BMDCS (Fig 5F).

#### FT-ICR-MS analysis indicates that FhTeg contains phosphosylated glycans, not sulphated

**Fig 6:**
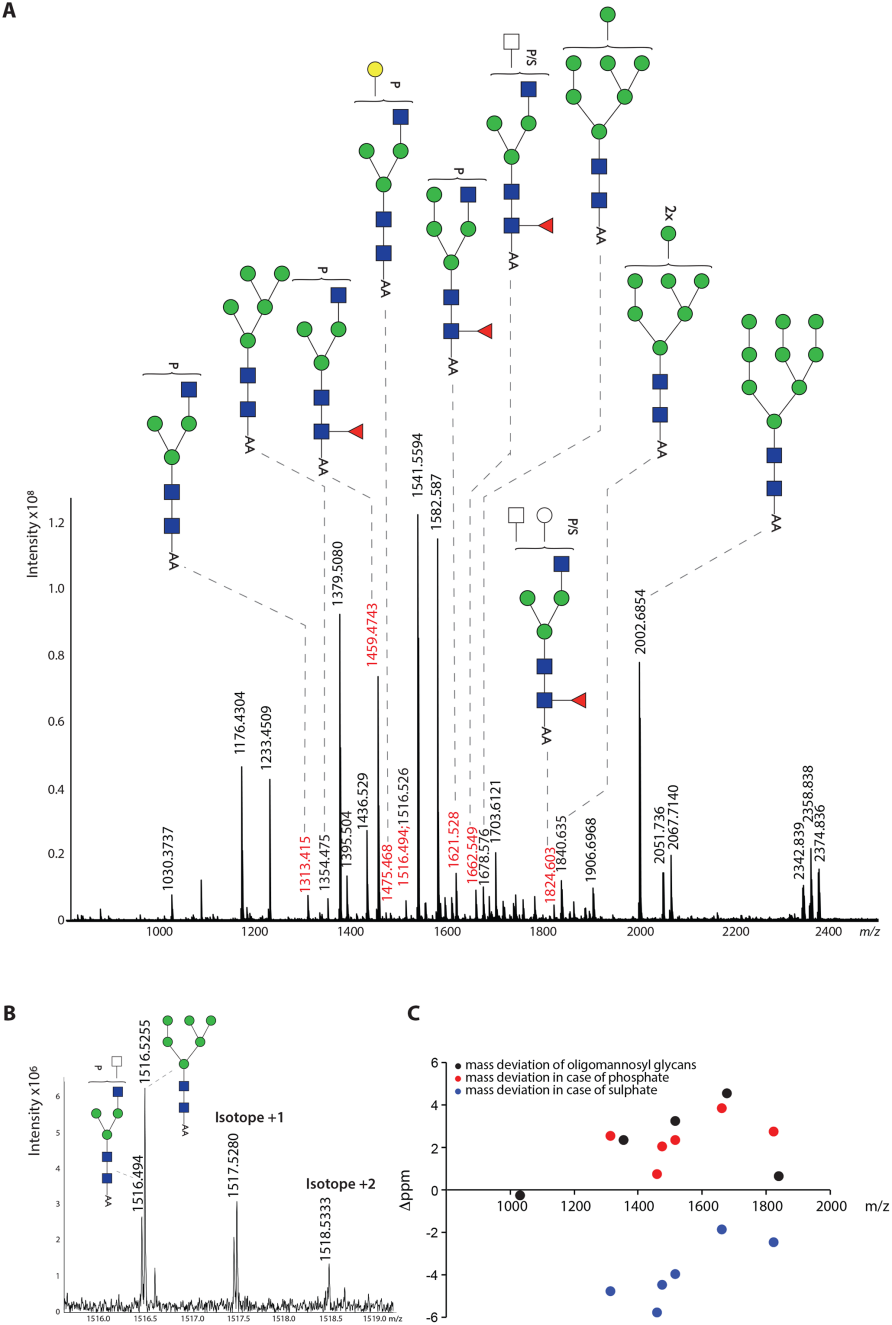
MALDI-FT-ICR-MS analysis of FhTeg N-glycans indicates the presence of phosphorylated glycan species. **A:** FT-ICR-MS spectrum indicating the phosphorylated glycans identified by accurate mass determination with external calibration and additional comparison with the internal confirmed oligomannose glycans. **B:** Zoom region showing the high resolution separation between the Man_6_GlcNAc_2_ glycan and a phosphorylated glycan of almost identical mass. **C:** The scatter plot indicates the deviation of the measured *m/z* from the calculated *m/z* of phosphorylated glycans in comparison with hypothetical sulphated glycans and the oligomannosyl glycans also present in the spectrum.

To clarify if the anionic glycan species detected at *m/z* 1313.6 and 1459.7 (Fig 1) carry a sulphate or a phosphate group a high-resolution MALDI-FT-ICR-MS spectrum was recorded (Fig 6A). Using the accurate mass of the mannosyl glycans for internal comparison, the phosphorylated glycans consistently fall in the same deviation range of 0 to +4 ppm, whereas the deviation of optional sulphated glycans would be distributed in the -2 to -6 ppm range (Fig 6C). This strongly indicates that the anionic glycans detected (Figs 1 and 2) contain phosphate, but not sulphate. Interestingly, the FT-ICR-MS analysis revealed the additional presence of a phosphorylated glycan species at m/z 1516.494 that was obscured in the conventional resolution MALDI-TOF-MS spectrum by the Man_6_GlcNAc_2_ glycan signal (m/z 1516.526 in Fig 6B, compare Fig 1).

### Anionic inhibitor GalNAc-4S and not Man-6P reverses FhTeg–induced upregulation of SOCS3 transcription levels in BMDCs

**Fig 7:**
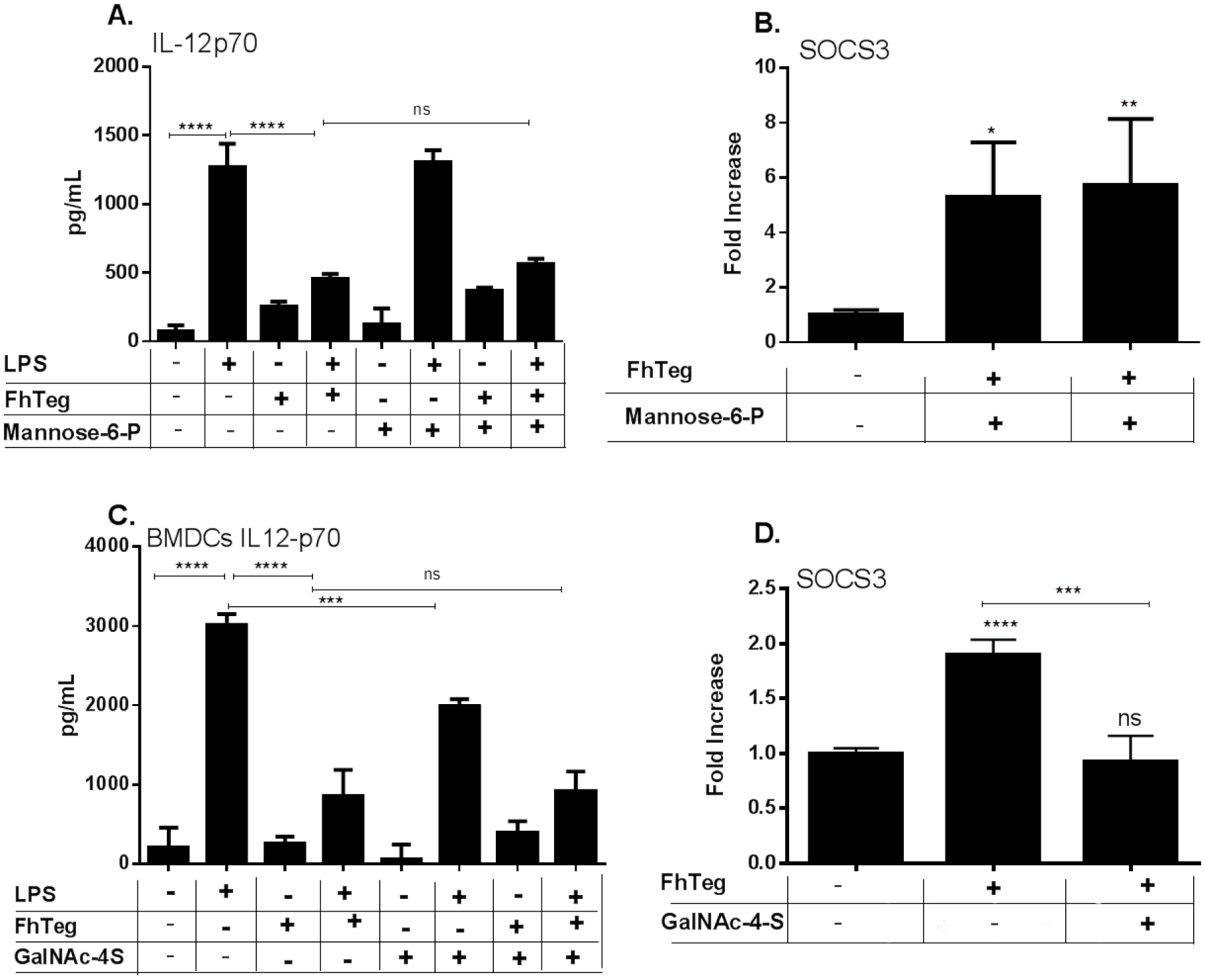
FhTeg–induced upregulation of SOCS3 transcription levels is reversed by anionic monosaccharide inhibitor GalNAc-4S and not Man-6P. **A,C:** BMDCs were pre-incubated with Man-6P (A) or GalNAc-4S (C) prior stimulation with PBS or FhTeg (10μg) before addition of LPS (100ng ml^-1^) for 18 h. IL12p70 levels were measured with commercial ELISA kits. Data are presented as the mean ± SEM of two independent experiments. ***p ≤ 0.001; ****p ≤ 0.0001 compared to LPS group. **B,D**: BMDCs were stimulated with Man-6P (B) or GalNAc-4S (D) for 30 min prior to incubation with FhTeg for 2.5 h. Total RNA was extracted, and after reverse transcription cDNA was analyzed with qPCR for SOCS3. RNA expression was normalized to GAPDH and actin control genes. **C**: BMDCs were stimulated with GalNAc-4S for 30 min prior to incubation with FhTeg for 2.5 h. Total RNA was extracted, and after reverse transcription cDNA was analyzed with qPCR for SOCS3. RNA expression was normalized to GAPDH and actin control genes.

In order to evaluate if the phosphorylated oligosaccharides contribute to the immunological properties of FhTeg, a panel of monosaccharide inhibitors, Man-6P, GlcNAc-4P and GalNAc-4S where investigated. Despite the absence of sulphated glycans on FhTeg, GalNAc-4S, a known inhibitor of the Cys-MR domain of the mannose receptor, was included in the panel as anionic control. Preincubation of BMDCs with Man-6P failed to suppress FhTeg immunological properties (Fig 7A and 7B). Notably, GlcNAc-4P interfered with culture media pH and expression level of housekeeping genes in CHO-MMR and BMDCs, respectively, resulting in unattendable results (S3 Fig). Interestingly, incubation with 1mM GalNAc-4S for 30 min prior to FhTeg stimulation failed to inhibit FhTeg ability to suppress the LPS-induced production of pro-inflammatory cytokines in BMDCs (Fig 7C) but reversed SOCS3 transcription to basal levels (p = 0.019), (Fig 7D). However the absence of sulphated glycans on FhTeg and the limited role played by MR in the immunomodulatory activity of the tegumental antigen suggest the involvement of a different CLR targeted by negatively charged glycoconjugates and inhibited by GalNAc-4S.

## Discussion

Host-pathogen interactions, immune cell development and function are mediated by glycans, glycolipidsand glycoproteins [48, 49] and in more recent years glycomics approaches have been used to facilitate the unravelling of these processes. In this study *N-*glycans were isolated and analysed by mass spectrometry for the first time from the tegumental antigen (FhTeg) of adult liver flukes to reveal that FhTeg-derived *N-*glycans are composed predominantly of oligomannose type and truncated structures. Mannosylated glycans are widely distributed on the tegument of adult flukes with remarkable abundance especially on the spines and suckers and are present on a wide panel of glycoprotein components of the FhTeg preparation. As these represent the fluke’s morphological features in most direct contact with the host, a role in cell adhesion and/or signalling for these glycoproteins is hypothesized. This study support previous findings by Allister et al. (2011) who using a panel of lectins examined the glycoprofile of *F*. *hepatica* gastrodermis that also exhibited an abundance of mannosylated glycoproteins [50].

Glycosylated antigens from *F*. *hepatica* excetory-secretory products and from other helminths (*S*. *mansoni*, *T*. *suis*, *T*. *crassiseps*, *T*. *spiralis*) have been shown to take part in immunomodulatory processes of parasitic infections *via* the mannose receptor (MR) [11,18,51,52,53]. MR is a type-I membrane protein with a cytoplasmic domain involved in antigen processing and receptor internalisation and three different types of binding domains at its extracellular region. In particular, MR features multiple C-type lectin-like carbohydrate-recognition domains (CRDs) responsible for Ca^2+^-dependent binding to terminal mannose, fucose or *N-*acetylglucosamine [54], a fibronectin type II (FNII) domain involved in collagen binding [55] and an *N-*terminal cysteine-rich (CR-MR) domain that mediates Ca^2+^-independent binding to sulphated sugars such as SO_4_-3-Gal or SO_4_-3/4-GalNAc [56]. In the case of helminth antigens, binding to MR has been always attributed to the interaction of antigen-derived glycans with the multiple CRDs that mediate MR binding to saccharides with terminal mannose, fucose or *N-*acetylglucosamine residues.

Here we show that blocking MR carbohydrate binding domains with specific glycan inhibitors (*i*.*e*. mannan for CRDs and GalNAc-4S for CR-MR) reversed the binding of FhTeg to BMDCs and CHO+MMR cells and a reduced binding was also observed in BMDCS isolated from MR knockout mice. However, it appears that MR plays a role in the binding of FhTeg to DCs but not in the signalling pathways, as FhTeg immune modulatory properties weren’t inhibited by preincubation with mannan *in vitro* and were retained in the absence of MR. These evidences suggest that other CLRs may play key roles in FhTeg immune modulation. Recently, the interaction of *F*. *hepatica* excretory-secretory products with macrophages demonstrated that MR and Dectin 1 blocking antibodies could inhibit the induction of M2 macrophages [18]. However, it has also been shown that TLR2 plays a role in the FhES inhibition of macrophages during *Mycobacterium bovis* activation [57*]*. *S*. *mansoni* soluble egg antigens have been shown to signal through several CLRs; DC-SIGN, MGL and MR to inhibit DC maturation and induce a Th2 immune response while the nematode *T*. *suis* antigens are involved in CLR signalling by inducing a DC phenotype which inhibits bacterial TLR activation and the activation of an inflammatory immune response [17]. Dectin 2 has also been implicated in the induction of IL-1β production from DCs following *S*. *mansoni* SEA stimulation [58]. The most extensively studied immunomodulatory glycans are conjugated and non-conjugated variants of the human milk sugar LNFPIII and its functional trisaccharide motif LeX that is expressed among many other glycans on various *schistosome* antigens [59]. In different forms LeX and/or LNFPIII induce M2-like macrophage [60], promote Th2 immune responses [61] and attenuate a range of inflammatory disorders including psoriasis [62] and transplant rejection [63]. While LeX binds to SIGNRI, this receptor does not have a role in its uptake by macrophages and it mechanism of action is TLR4 dependent [64]. LeX is not found in *F*. *hepatica* but in general helminth antigens are composed of a complex mix of glycans, proteins and lipids, and it is reasonable to assume that multiple TLR and CTRs are involved in its immune properties with a number of redundancy pathways.

The unexpectedly limited role played by the mannose receptor in FhTeg mechanism of immunomodulation prompted a further mass spectrometric investigation to elucidate the nature of other terminal sugars. The most notable aspect of the MS spectrum of FhTeg-derived *N-*glycans was the discovery of structures modified with anionic groups on terminal residues. Negatively charged glycans on the glycocalyx of adult liver flukes have been previously observed by electron microscopy when staining acid carbohydrates with cationic dyes such as colloidal iron, ferric chloride or ruthenium red [31]. In his pioneering study on *F*. *hepatica* tegument, Threadgold could not investigate the nature of the anionic groups due to the lack of adequate experimental tools. FT-ICR-MS was conducted on FhTeg-derived N-glycans and unequivocally ascertained the presence of phosphate and not sulphate groups on terminal Man or GlcNAc monosaccharides.

Interestingly, an acidic glycolipid fraction containing the highly antigenic phosphodiester, GlcNAc(α1-HPO3-6)Gal(1–1)ceramide, has been previously reported in *F*. *hepatica* [35]. In this study, MS/MS fragmentations suggest that, differently from the glycolipid, in FhTeg the phosphate group is bound terminally as monoester to a single monosaccharide (Man or GlcNAc). Notably, phosphorylation of N-glycans is predominantly found on high-mannose type glycans in the form of Man-6P monoester or as phosphodiester capped by α-GlcNAc (i.e. GlcNAc-P-Man). These phosphorylated glycan epitopes are responsible for the targeted trafficking of hydrolases to lysosomes through recognition by P-type lectins, CI-MPR and CD-MPR [65]. The presence of parasitic lysosomal hydrolases in FhTeg antigen preparation cannot be excluded, but it has to be noted that Man-6-phosphorylation has also been reported on non-lysosomal proteins [66,67,68,69]. Interestingly, Man-6-phosphorylation is observed on complex-type N-glycans of envelope glycoproteins of varicella zoster virus involved in viral entry [70,71,72]. Scattered reports have described the presence of phosphorylation on monosaccharides other than mannose, however no clear understanding of its role has emerged thus far [73,74,75]. In particular, in the case of the synapse-specific clathrin assembly protein AP180, the presence of two sites of O-linked glycosyl phosphorylation (GlcNAc-6P) contributes to an increased protein net negative charge and hydrophilicity, suggesting a potential inhibitory effect in synaptic vesicle endocytosis processes [75].

In this study, the nature and the potential biological role played by these negatively charged glycoconjugates was further exploredsince oligosaccharide phosphorylation hasn’t been observed in any other helminth for which glycomics investigations have been carried out thus far,. Notably, inhibition assays revealed that some key immune modulatory properties exhibited by FhTeg are independent from Man-6P, thus excluding a potential role for Mannose 6-Phosphate Receptor in *Fasciola* infection mechanism. FhTeg-induced expression of SOCS3 was reversed upon pre-incubation of DCs with GalNAc-4S, however this did not reverse the immune modulatory properties of FhTeg suggesting that SOCS3 despite been induced during infection may not be important to its immune modulatory properties. However further studies are required to confirm this while other negative regulators of cytokines signaling such as SOCS2 have yet to be examined. GalNAc-4S was originally included in this study merely as a control to explore the anion specificity of the inhibition mechanism and the effect upon FhTeg activated DCs was not expected as sulphated anionic glycans were not identified in FhTeg and the role of MR was excluded in these studies. A possible explanation is that many CLRs can interact with more than one type of glycan structure and the addition of GalNAc-4S to our assays may give insight into the type of receptor but not the type of sugar important in this process. However, to date evidence supports the binding of GalNAc-4S to the CD of MR suggesting that this glycan may bind to an alternative receptor.

While our data points towards a possible new pathway linking SOCS3 expression and CLR stimulation we have yet to identify the exact receptor. This is supported by work by Rolls et al. (2006) that show increased SOCS3 expression and decreased secretion of IFN-γ and TNF in T cells stimulated with chondroitin sulfate proteoglycan (CSPG-DS) *in vitro* [76]. No cellular receptor has been identified yet for this antigen. SOCS3 was also found to be induced in immature DCs as result of DC-SIGN stimulation by HIV-1 envelope glycoprotein gp120 thorough a complex pathway involving other factors such as Ras, Raf, and NF-κB-induced IL-10 secretion [77]. This supports our observation that SOCS3 induction through CLRs stimulation by glycoconjugates could be a driving mechanistic step in this process [78,79] but the exact receptor needs to be identified.

In summary, while this study suggests a role for CLRs in FhTegs interaction with BMDCs, MR is not directly involved in the immune modulatory properties measured in the parameter of this study. While we have not identified the receptor involved in the induction of SOCS3 or in the suppression of pro-inflammatory cytokine, we believe that given the spectrum of *N*-glycans identified in FhTeg a variety of pathways may be activated simultaneously in a glycan-dependant manner by the heterogeneous mixture of glycoproteins that compose FhTeg. Further studies and a simplification of the tegumental antigen are required to fully dissect the effect of individual components. The discovery of phosphorylated *N-*glycans in FhTeg flukes highlight a potential a novel role for these molecules during helminth infection.

## Supporting Information

S1 Fig MALDI-TOF-MS of α-mannosidase (A), β-hexosaminidase (B), β-galactosidase (C) and neuraminidase (D) digested AA-labelled *N-*glycans from Fh tegumental preparation. Aliquots of FhTeg 2-AA-labeled-*N*-glycans were incubated with Jack bean α-mannosidase (Sigma-Aldrich) in NaAc pH 4.5, 100mM NaAc pH 5, Jack bean β-(1–4,6) galactosidase (Prozyme-Glyko), β-*N-*acetylglucosaminidase from *Canavalia ensiformis* or neuraminidase from *Vibrio cholera* (Sigma-Aldrich) in the provided reaction buffers for 16 h at 37°C and analyzed by MALDI-TOF-MS (in the negative ion-reflectron mode) after application to Zip-Tip C18 and direct elution onto the target plate with a solution of DHB in 30% ACN. Signals are labelled with monoisotopic masses. Panel D is presented as overlay of MALDI-TOF-MS spectra detail of natural-occurring and neuraminidase digested AA-labelled *N-*glycans from Fh tegumental preparation.
(TIF)

S2 Fig The spectrum of PNGase A released glycans: Fh tegumental antigens were digested with trypsin followed by PNGase A treatment. Released N-glycans were subsequently labelled with 2-AA and analysed by MALDI-TOF-MS in the negative ion-reflector mode. Signals are labelled with monoisotopic masses. Most abundant *N*-glycan structures are annotated in the spectrum while minor peaks are reported in the supplementing material (S1 Table). The signal at *m/z* 1582.8 [M-H]^-^ is annotated according to MALDI-TOF/TOF-MS analysis.
(TIF)

S3 Fig Pre-incubation of BMDCs with GlcNAc-4P interfered with expression level of housekeeping genes. BMDCs were stimulated with and without GlcNAc-4P (1mM), for 45 min prior to stimulation with FhTeg. Total RNA was extracted, and after reverse transcription cDNA was analyzed with qPCR for SOCS3. RNA expression was normalized to GAPDH and actin control genes.
(TIF)

S1 Table Glycan signal (m/z) and proposed structures. (TIF)
