# Supplementary figures and images for: Fasciola hepatica Surface Coat Glycoproteins Contain Mannosylated and Phosphorylated N-glycans and Exhibit Immune Modulatory Properties Independent of the Mannose Receptor
Source: PLoS Negl Trop Dis. 2016 Apr 22;10(4):e0004601. doi: 10.1371/journal.pntd.0004601 (PMC4841591; doi:10.1371/journal.pntd.0004601)

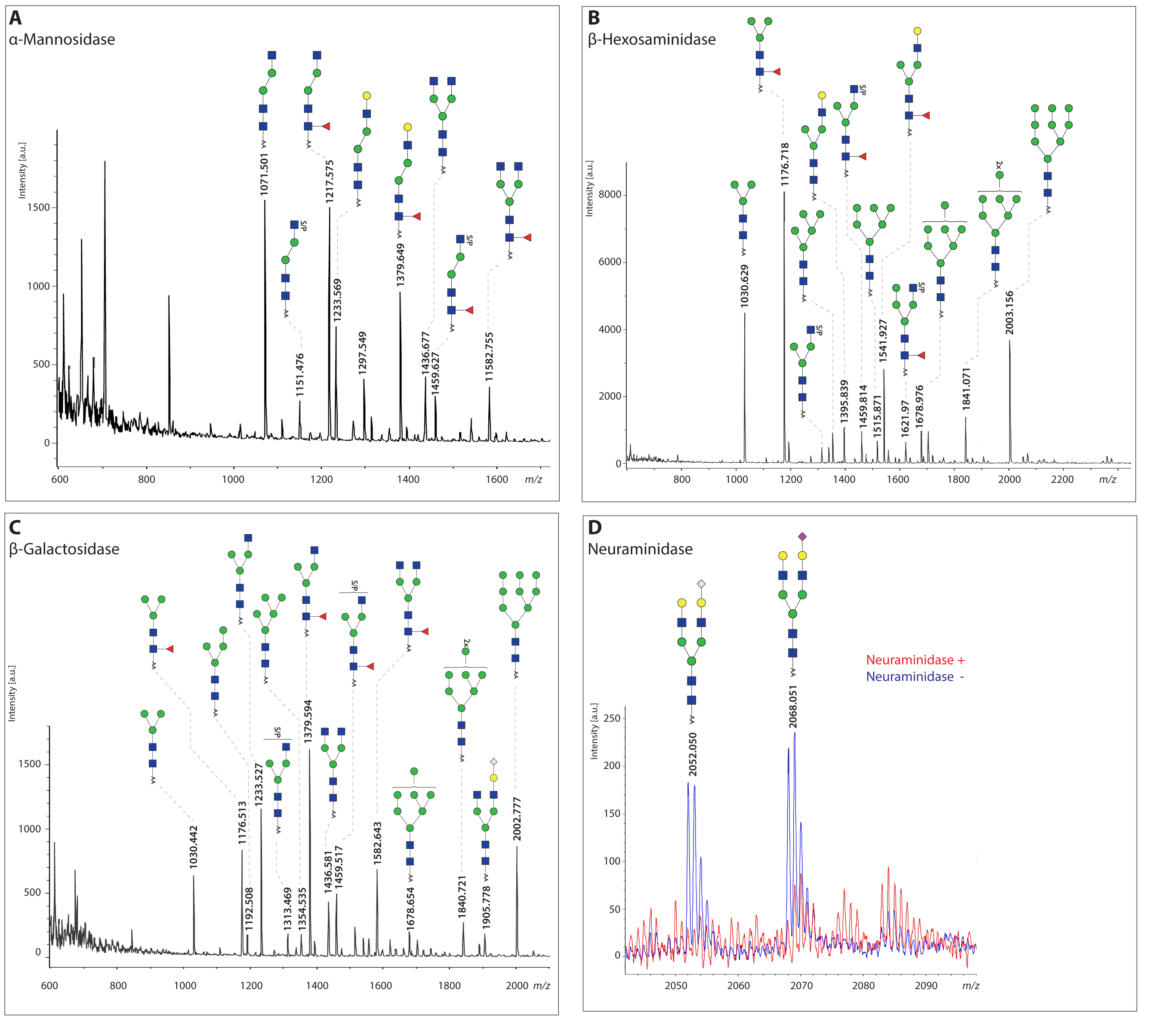

Supplement: S1 Fig — Aliquots of FhTeg 2-AA-labeled-N-glycans were incubated with Jack bean α-mannosidase (Sigma-Aldrich) in NaAc pH 4.5, 100mM NaAc pH 5, Jack bean β-(1–4,6) galactosidase (Prozyme-Glyko), β-N-acetylglucosaminidase from Canavalia ensiformis or neuraminidase from Vibrio cholera (Sigma-Aldrich) in the provided reaction buffers for 16 h at 37°C and analyzed by MALDI-TOF-MS (in the negative ion-reflectron mode) after application to Zip-Tip C18 and direct elution onto the target plate with a solution of DHB in 30% ACN. Signals are labelled with monoisotopic masses. Panel D is presented as overlay of MALDI-TOF-MS spectra detail of natural-occurring and neuraminidase digested AA-labelled N-glycans from Fh tegumental preparation. (TIF) [file pntd.0004601.s001.tif]

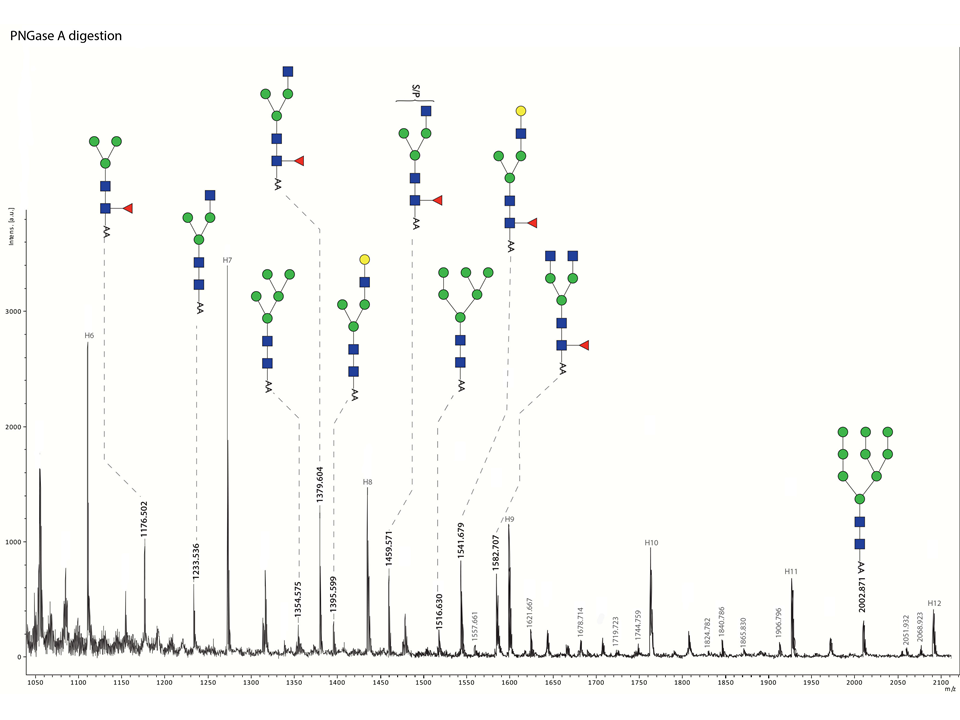

Supplement: S2 Fig — Released N-glycans were subsequently labelled with 2-AA and analysed by MALDI-TOF-MS in the negative ion-reflector mode. Signals are labelled with monoisotopic masses. Most abundant N-glycan structures are annotated in the spectrum while minor peaks are reported in the supplementing material (S1 Table). The signal at m/z 1582.8 [M-H]- is annotated according to MALDI-TOF/TOF-MS analysis. (TIF) [file pntd.0004601.s002.tif]

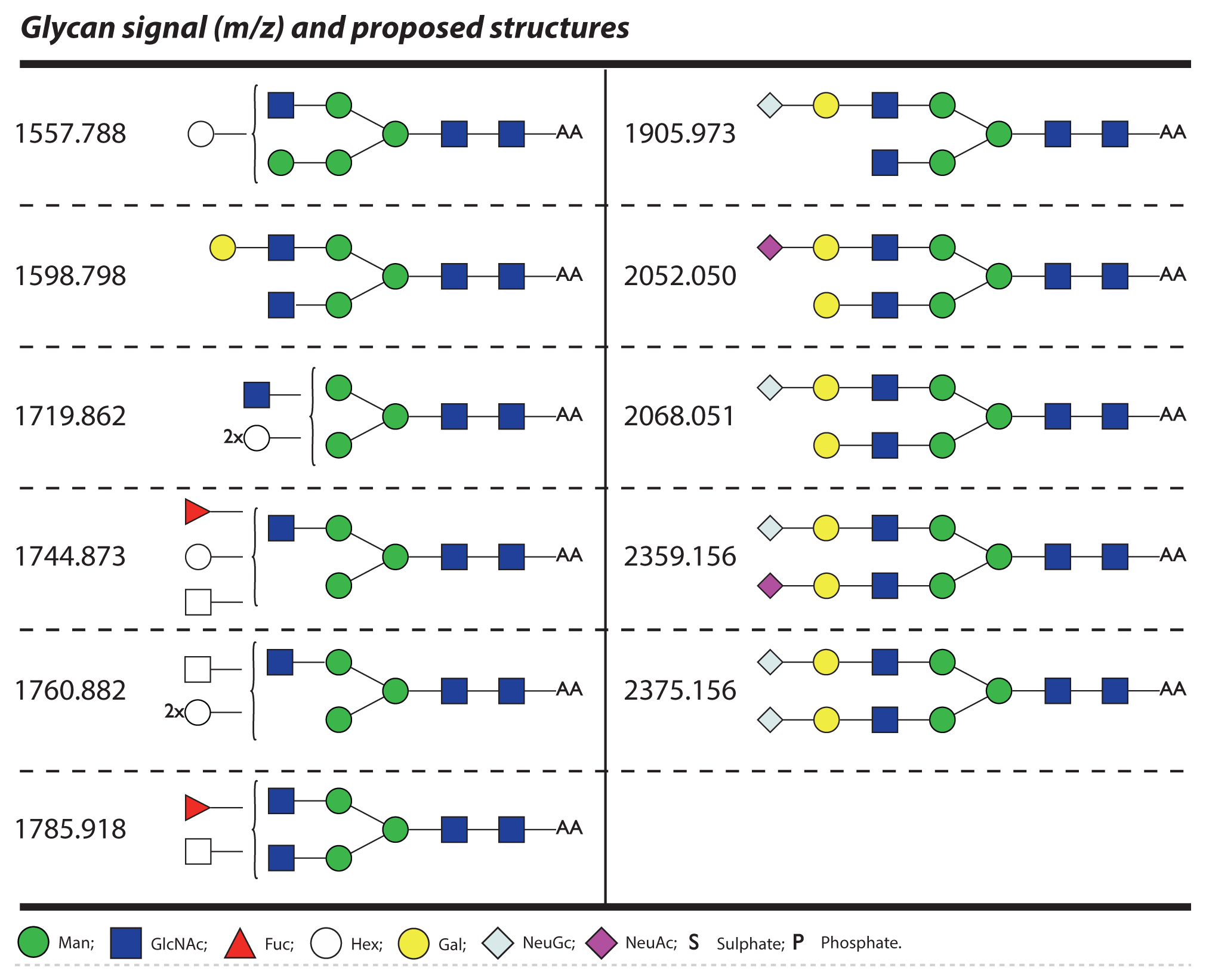

Supplement: S1 Table — (TIF) [file pntd.0004601.s004.tif]
